# Leprosy on the scalp*

**DOI:** 10.1590/abd1806-4841.20164391

**Published:** 2016

**Authors:** Raila de Brito Macedo, Tárcio Santos, Paulyane Bezerra Sampaio Ramos, Daniela Mayumi Takano, Virgínia Sampaio Madeiro Leal

**Affiliations:** 1 Centro de Estudos Dermatológicos do Recife (CEDER) - Recife (PE), Brazil; 2 Universidade Federal de Pernambuco (UFPE) - Recife (PE), Brazil; 3 Private clinic - Recife (PE), Brazil

**Keywords:** Scalp dermatoses, Hansen’s disease, *Mycobacterium leprae*

## Abstract

Leprosy is a chronic infectious disease caused by *Mycobacterium leprae*. This bacillus has a high predilection for skin and peripheral nerves. The scalp’s anatomical properties do not favor the development of such mycobacterium. We report a case of leprosy with scalp involvement, a rare occurrence in our literature.

## INTRODUCTION

Leprosy is endemic in Brazil, ranking second in the absolute number of cases worldwide, topped only by India.^1,2^ In humans, the bacillus enters through the upper airways and lodges in the branches of cutaneous nerves and peripheral nerve trunks.^2^ Several classifications have been proposed for this disease. Ridley & Jopling’s (1962, 1966) focus on the spectral concept of leprosy and is based on clinical criteria, microscopy, immunology, and histopathology. The extreme forms of the spectrum are polar tuberculoid (TT) leprosy and polar lepromatous (LL) leprosy, which are further classified into dimorphic-tuberculoid (DT) leprosy, borderline-lepromatous (BL) leprosy, and dimorphic-dimorphic (DD) leprosy.^3^

The patient’s clinical evolution depends on the number of bacilli present and the host immunopathological response.^3,4^ Recognition of clinical forms and early diagnosis are key to disease control. According to current regulations, recognition of leprosy must be primarily clinical.^5^

The low occurrence of alopecia may explain the apparent rarity of scalp involvement. Also, the scalp’s anatomical structures may obscure the prominence of the lesions so that they can not be easily detected.^6,7^ Several years ago authors did not think that the mycobacteria affected the scalp; however, with new histopatological techniques, this is now widely accepted.^6,7^

## CASE REPORT

**Figure 1:**
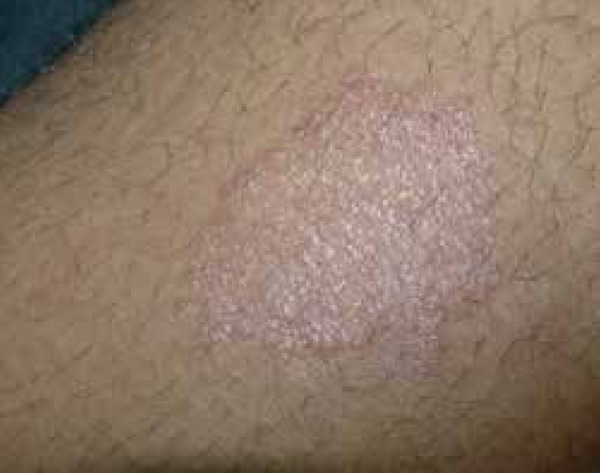
Well-defined erythematous infiltrated plaque on the left thigh

**Figure 2:**
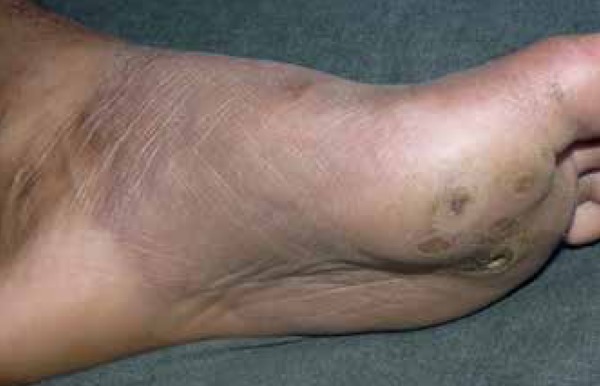
Well-defined erythematous infiltrated plaque with centrifugal growth on the cavus and left plantar region

**Figure 3:**
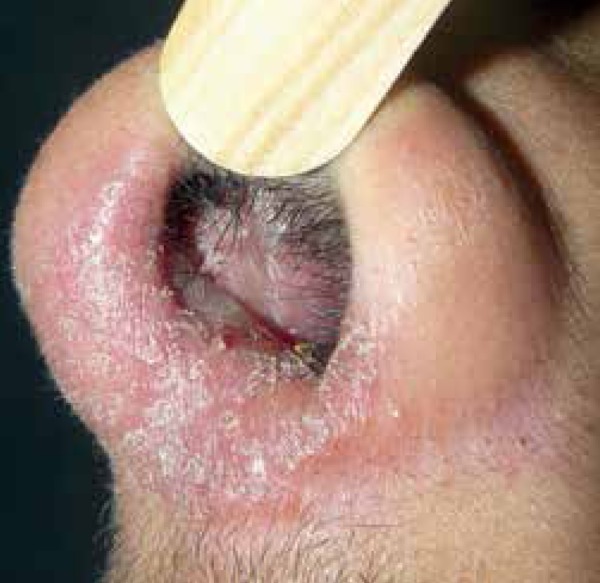
Well-defined erythematous infiltrated plaque on the columella

**Figure 4:**
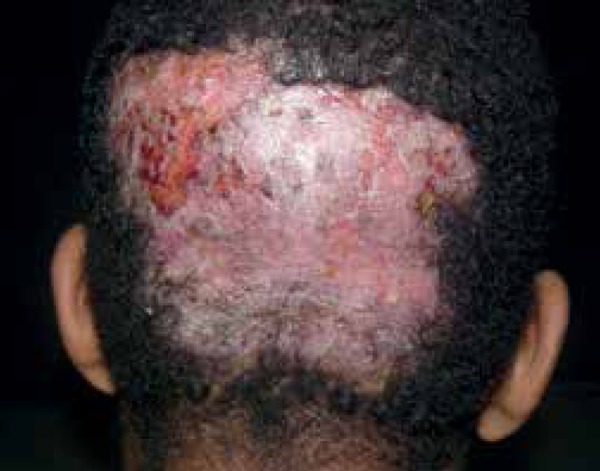
Well-defined infiltrated erythematous alopecia plaque on the scalp, occipital segment

An 18-year-old brown-skinned male student, born and raised in Olinda, Brazil reported the occurrence of well-defined lesions on the left foot and thigh a year and four months before the report. The lesions showed centrifugal growth and no associated symptoms (Figures 1 and 2). The disease progressed with the appearance of itchy lesions on the scalp and columella (Figures 3 and 4). He denied similar events in his family or comorbidities.

**Figure 5:**
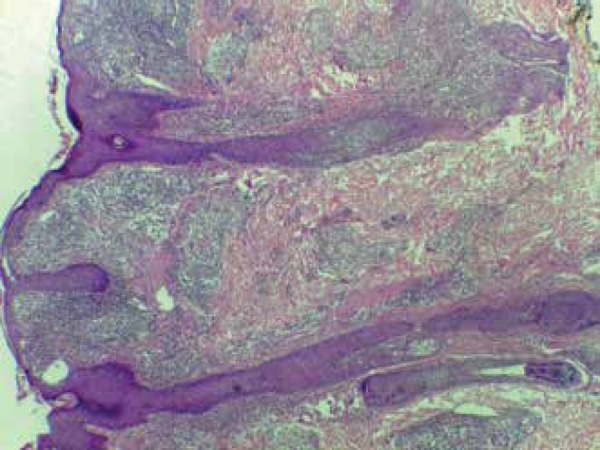
Histopathology revealing lymphohistiocytic inflammatory infiltrate on the dermis, with granuloma formation, spreading aroundthe neurovascular plexus and skin appendages

Clinical examination of the integument showed 5 infiltrated erythematous scaly plaques with well-defined borders, except for the lesion on the scalp. The scalp showed an infiltrated plaque of alopecia with bullous and other areas with meliceric crusts. Thermal and pain testing ware performed, and both showed changes for all lesions. Mycological testing with direct examination and culture were both negative. A biopsy of the scalp lesion was performed, and histopathology showed dermis involvement by lymphohistiocytic inflammatory infiltrate with multinucleate giant cells. We also observed epithelioid granulomas spread around the neurovascular plexus and skin appendages (Figure 5). We performed an AFB test with Ziehl-Neelsen stain; both biopsy and lymph materials were negative.

**Figure 6:**
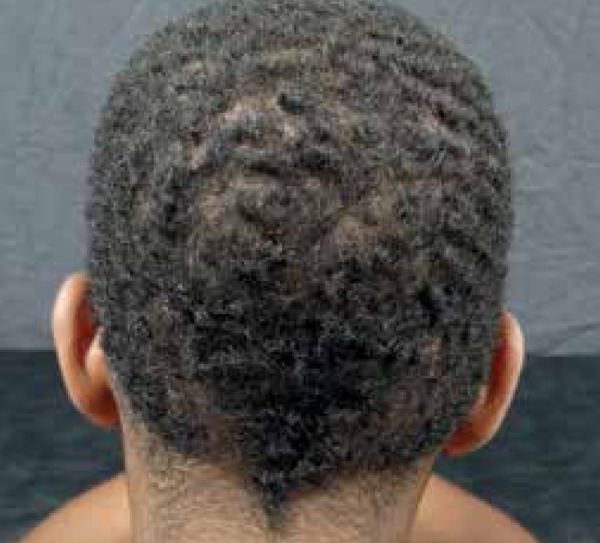
Absence of lesion after treatment completion

After multidrug therapy for multibacillary leprosy, we observed integument lesion resolution, scalp hair regrowth, and improved paresthesia (Figure 6).

## DISCUSSION

*Mycobacterium leprae* prefers colder areas of the human body.^6,7^ As the scalp has higher temperatures, this bacterium usually avoids this location. A few cases of leprosy involving the scalp, especially the frontal region and in patients with lepromatous leprosy have been reported.^6^

Besides temperature, other anatomical characteristics of the scalp hinder the spread of inflammatory infiltrates in this area.^6^ One example is the tension system among the cleavage lines, subcutaneous tissue, aponeuroses, and muscles.^6^ Tension lines are particularly rich in adipose tissue, and the fat lobes are compressed by fibrous septa along the dermis and aponeurose.^6^ Infiltration, papules, and nodules are the most common lesions when the hair is intact. Alopecia secondary to leprosy is mild and unusual.^6^

Fully developed hair is located in the subcutaneous tissue.^7^ Studies on leprosy with scalp involvement with mild and moderate infiltration showed no impairment of the vital part of the follicle as the deeper areas are not affected.^6^ In this case, patients with senile and Hippocratic alopecias would be more likely to have their hair follicles – which are in regression – affected by the inflammatory infiltrate. Therefore, alopecia secondary to leprosy is more likely to occur in patients with Hippocratic and senile alopecia due to the predominance of superficial follicles.^7^

The present case report draws attention to the dimorphic- tuberculoid characteristic of the disease in an 18-year-old patient who reported no androgenetic alopecia but developed alopecia secondary to leprosy.
